# Enzymatic Hydrolysis of Porcine Blood as a Strategy to Obtain a Peptide-Rich Functional Ingredient

**DOI:** 10.3390/ijms26209863

**Published:** 2025-10-10

**Authors:** Cristina Moreno-Mariscal, Federico Moroni, Jaume Pérez-Sánchez, Leticia Mora, Fidel Toldrá

**Affiliations:** 1 Instituto de Agroquímica y Tecnología de Alimentos (IATA-CSIC), Avenue Agustín Escardino 7, 46980 Paterna, Spain; cmormar@iata.csic.es (C.M.-M.); ftoldra@iata.csic.es (F.T.); 2 Fish Nutrigenomics and Integrative Biology Group, Instituto de Acuicultura Torre de la Sal (IATS-CSIC), 12595 Ribera de Cabanes, Spain; jaime.perez.sanchez@csic.es

**Keywords:** enzymatic hydrolysis, bioactive peptides, porcine blood, aquaculture, functional ingredients, functional feed

## Abstract

The sustainable revalorization of porcine blood is crucial due to the large volumes daily generated in slaughterhouses. The aim of this study was to obtain a novel ingredient rich in free amino acids and bioactive peptides from the sequential hydrolysis of porcine blood. Porcine blood was hydrolyzed with Alcalase 4.0 L and Protana™ Prime enzymes, followed by molecular weight fractionation (<10 kDa) and spray-drying. The antioxidant, hypoglycemic, and anti-inflammatory bioactivities of the resulting hydrolysate (PBSH) were studied in vitro. Further fractionation by reversed-phase high-performance liquid chromatography (RP-HPLC) was performed to isolate the most bioactive fraction based on polarity. Peptides from fraction 1 (F1) were identified using LC-MS/MS and analyzed in silico. Finally, some peptides were synthesized, and their bioactivity was subsequently assessed. PBSH hydrolysate showed antioxidant activity with IC_50_ values of 2.09, 135.05, and 26.73 mg/mL for ABTS, FRAP, and DPPH assays, respectively. Additionally, PBSH exhibited hypoglycemic, anti-inflammatory, and immunomodulatory potential through the inhibition of DPP-IV (82.78%), NEP (84.72%), TACE (50.79%), and MGL (69.08%) enzymes at a concentration of 20, 20, 100, and 20 mg/mL, respectively. Peptides PDDFNPS, FPPKPKD, DNPIPK, GHLDDLPG, and GDL were identified in the most polar and bioactive fraction (F1) and proved a synergistic hypoglycemic effect at a concentration of 1 mmol/L. The peptide PDDFNPS exhibited multifunctional properties with 56.43% inhibition of DPP-IV and 83.54% inhibition of NEP. PBSH resulted in a novel functional ingredient for animal feed as it contains a variety of identified bioactive peptides and a high amount of free amino acids.

## 1. Introduction

Growing food demand has largely increased the generation of by-products, and currently more than 20 million tons of animal by-products (ABPs) (including skin, bones, blood, feathers, etc.) are produced annually in the European Union (EU) from slaughterhouses, farms, and the food industry [1]. The EU is the second largest producer of pig meat in the world after China, but also the largest exporter, with approximately the 13% of the production going to the East of Asia [2]. As a result, valorization of porcine by-products has become an active area of research, driven by the need for sustainable practices for both food and animal feed production. In this regard, it must be noted that blood is one of the main by-products generated in slaughterhouses, representing up to 3.0–3.5% of the live weight of pigs [3]. In some countries, hygienically collected blood is used to produce different traditional meat products such as blood sausages or puddings [4]. Otherwise, pig blood is often used as an ingredient in fertilizers [5] but also as a protein source of high biological value (20%) in animal feeds, particularly in aquafeed, rendering different technological properties, such as water retention and gel formation and emulsifier properties [3,6].

The above considerations also apply to the aquaculture sector, which is considered the fastest-growing animal production sector over the last twenty years [7]. However, the future development of this sector is limited by its heavy reliance on finite marine resources, particularly fishmeal and fish oil, which are derived from wild-caught forage fish [8]. This dependence exerts pressure on natural fish stocks, drives price volatility, and raises concerns regarding long-term ecological sustainability and food security [9]. To maintain growth while reducing environmental impact, this sector must change to alternative feed ingredients including insect proteins, microbial biomass, and processed animal proteins (PAPs) derived from the valorization of meat industry by-products such as poultry meat meal, feather meal, whole dried blood, and plasma proteins [10,11,12]. Enzymatic hydrolysis is perhaps the most common process for the revalorization of such ABPs [13,14,15,16], being devoted to the use of protein hydrolysates as a novel feed ingredient not only to enhance the sustainability and efficiency of the aquaculture sector but also to promote a circular economy within the meat industry by reducing food waste and adding value to their by-products [17,18]. Accordingly, enzymatic hydrolysis is considered the most effective and sustainable alternative for the revalorization of livestock blood, allowing the obtaining of extracts rich in free amino acids (FAAs) and biologically active peptides [19,20], which in turn improves protein digestibility and bioaccessibility but also prevents potential allergies [21]. This versatility makes protein hydrolysates excellent functional ingredients for incorporation into animal feeds.

Recently, we optimized the sequential enzymatic hydrolysis of porcine blood to obtain extracts rich in bioactive peptides with in vitro antioxidant, hypoglycemic, and anti-inflammatory activities [22]. One of the resulting hydrolysates, obtained with the endopeptidase Alcalase 4.0 L and the exoprotease Protana™ Prime, exhibited a larger amount of bioactive peptides with non-allergenic and non-toxic properties in comparison to hydrolysates generated by other enzymatic combinations. Moreover, this extract obtained by Alcalase 4.0 L and Protana™ Prime also showed a significant hypoglycemic potential in vitro, exhibiting the highest neprilysin (NEP) inhibitory activity among the tested porcine blood hydrolysates.

The aim of this study was to obtain a novel functional ingredient rich in bioactive peptides and free amino acids through the sequential hydrolysis of porcine blood with Alcalase 4.0 L and Protana™ Prime enzymes, followed by the characterization of its bioactivity and peptide identification. This research provides an innovative approach to obtain functional ingredients from animal by-products for application in animal feed. It promotes the circular economy and reduces dependence on natural resources for feed formulation, principally in the aquaculture sector.

## 2. Results

**Figure 1:**
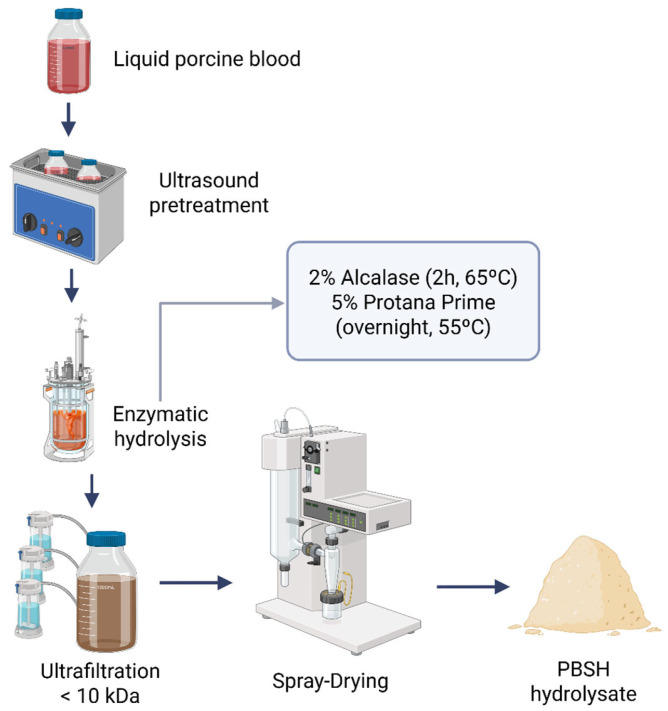
Flow diagram of the PBSH hydrolysate production process (Created in https://BioRender.com, accessed on 10 February 2025).

The spray-dried hydrolysate PBSH (Figure 1), containing 86.24% of protein according to the Dumas method, was concentrated by ultrafiltration using 10 kDa membrane filters and later characterized. Regarding this, it has been widely described that bioactive sequences used to be between 2 and 20 amino acids in length, so the isolated fraction was expected to be rich in small peptides and free amino acids that could exert a biological action [23].

### 2.1. Biological Activities of Porcine Blood Spray-Dried Hydrolysate (PBSH)

ABTS radical scavenging activity, ferric-reducing antioxidant power (FRAP), and DPPH radical scavenging activity assay are three methods based on the electron-donating capacity of antioxidant compounds. As shown in Table 1, the hydrolysate (PBSH) presented high antioxidant values for the three assayed methodologies [24], probably due to the presence of low molecular size peptides. Positive controls resulted in 2263.31 ± 1.90 µmol TEAC/g, 1183.93 ± 13.15 µmol TE/g, and 91.30 ± 0.38% values for the ABTS, FRAP, and DPPH assays, respectively. However, the best results were obtained in the ABTS assay, with an IC_50_ of 2.09 mg/mL, while the FRAP and DPPH assays resulted in values of 135.05 and 26.73 mg/mL, respectively.

The stability study of antioxidant activity (Table 2) showed non-significant differences in the ABTS assay during a storage period of six months under refrigerated conditions, whereas, at room temperature, PBSH hydrolysate exhibited significant changes along the time of storage, notably due to the instability of the extract under these conditions. In the FRAP assay, the activity was more unstable, with better preservation under refrigeration. At room temperature, the activity increased steadily, probably due to the degradation of large peptides into smaller peptides, which could enhance the antioxidant activity. In the DPPH assay, there were non-significant differences during the first five months under refrigeration, although a progressive increase in antioxidant activity was observed at room temperature.

Finally, the PBSH hydrolysate exhibited strong inhibitory effects on DPP-IV (82.78%), NEP (84.72%), TACE (50.79%), and monoacylglycerol lipase (69.08%), as shown in Table 1.

### 2.2. Free Amino Acid Composition of PBSH Sample

As is shown in Table 3, the PBSH showed an amount of 461.82 mg of free amino acids /g of sample, which represents almost half (46%). This is probably due to both the strong exoprotease activity of Protana Prime, with high capacity to release amino acids, and the membrane filtering concentration to <10 kDa of the extract. 

This hydrolysate contains the nine essential amino acids, with a high content of leucine (around 18%), valine (11%), lysine (10%), histidine (8.7%), and phenylalanine (7.3%), which represent almost 40% of the total amino acids amount.

### 2.3. Separation and Characterization of PBSH Fractions

**Figure 2:**
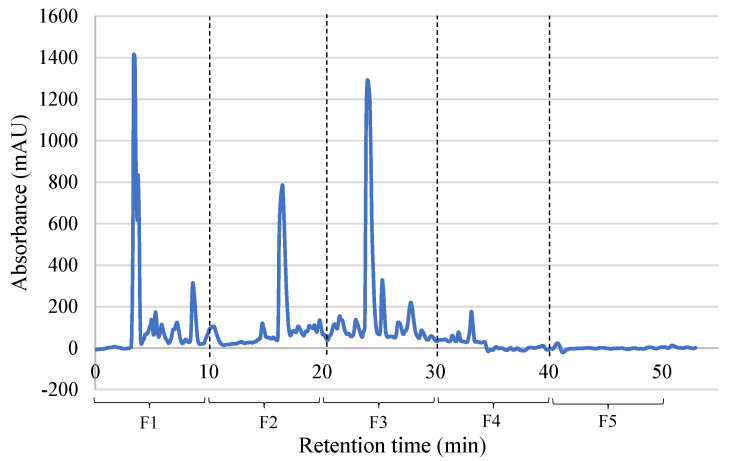
Elution profile of the porcine blood spray-dried hydrolysate (PBSH) obtained after RP-HPLC chromatography.

PBSH hydrolysate was subjected to a separation process by RP-HPLC according to the polarity of its peptides (Figure 2), with firstly the most polar and later the most hydrophobic peptides being eluted. The isolated fractions were analyzed to test the biological activities, as shown in Table 4.

#### 2.3.1. Biological Activities of PBSH Fractions Obtained by RP-HPLC

Regarding antioxidant activity using the ABTS assay, fraction 2 (F2) showed a value of 1441.31 µmol TEAC/g, which was significantly higher than the other fractions, followed by fraction 3 (F3) with 1397.22 µmol TEAC/g. However, in the FRAP and DPPH assays, fraction 1 (F1) showed the highest results with 89.94 µmol TE/g and 17.01%, respectively (Table 4).

Regarding hypoglycemic activities, the fractions also showed DPP-IV and NEP inhibitory activity. Thus, F1 showed the highest inhibition values for both enzymes with percentages of 90.45% for DPP-IV and 84.69% for NEP. Regarding NEP inhibitory activity, a decrease with increasing fraction number was observed, probably due to the changes in the polarity of peptides. In DPP-IV inhibitory activity, this trend was not clear, since F2 and F3 showed no activity, and the activity of F4 was 55% lower than F1.

The polarity of the peptides also affected the inhibitory activity of the TACE enzyme, since a progressive decrease in activity was also observed as the peptides became more hydrophobic. Finally, only F4 was able to inhibit the MGL enzyme, with an inhibition value of 85.30%.

#### 2.3.2. Protein and Peptide Identification of PBSH Fractions by LC-MS/MS

**Figure 3:**
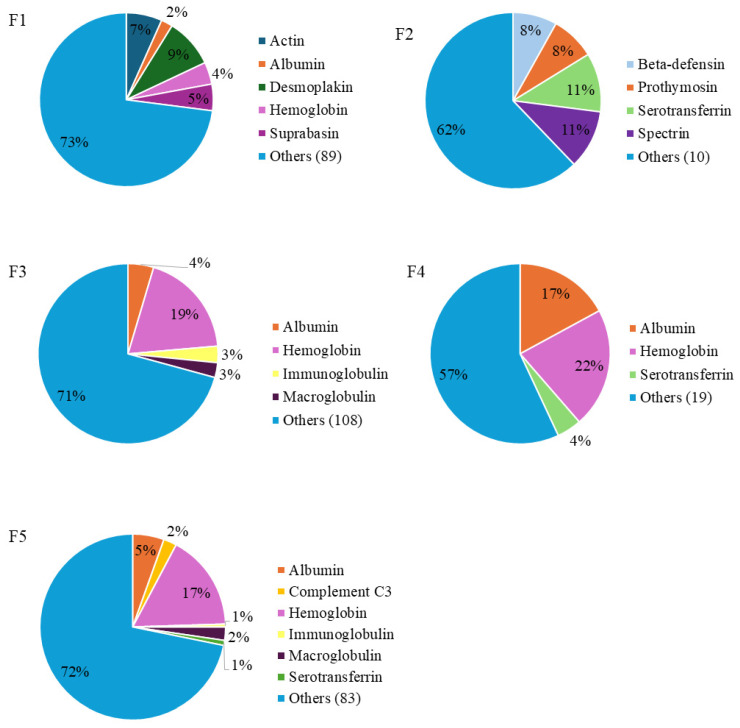
Protein distribution of the most relevant blood proteins according to the total number of peptides identified by LC-MS/MS in porcine blood spray-dried hydrolysate (PBSH) fractions. The results are represented as a percentage of peptides from each protein out of the total number of unique peptides identified.

LC-MS/MS analysis of the PBSH HPLC fractions allowed the identification of a total of 4524 peptides. As shown in Figure 3, there were differences in the distribution of peptides according to their protein of origin. The main protein sources were proteins from blood, such as albumin, hemoglobin, immunoglobulin, and serotransferrin.

In F1, which proved to be the most bioactive, a total of 1439 unique peptide sequences were identified, coming from a wide variety of proteins. A total of 9% of the identified sequences were from desmoplakin, which is a key protein for maintaining cell adhesion in cardiomyocytes [25], followed by actin protein with 7%, and hemoglobin with 4%. On the other hand, a total of 37 sequences were identified in F2, mainly from serotransferrin (11%) and spectrin (11%) proteins. In F3, F4, and F5, hemoglobin was the main source of peptides, with percentages of 19, 22 and 17%, respectively. The presence of peptides derived from albumin was also important in these fractions, constituting 17% of the total peptides in F4, and there was also a large number of peptides derived from immunoglobulins, serotransferrin, or macroglobulin proteins.

### 2.4. Characterization of Most Active HPLC Fraction 

**Figure 4:**
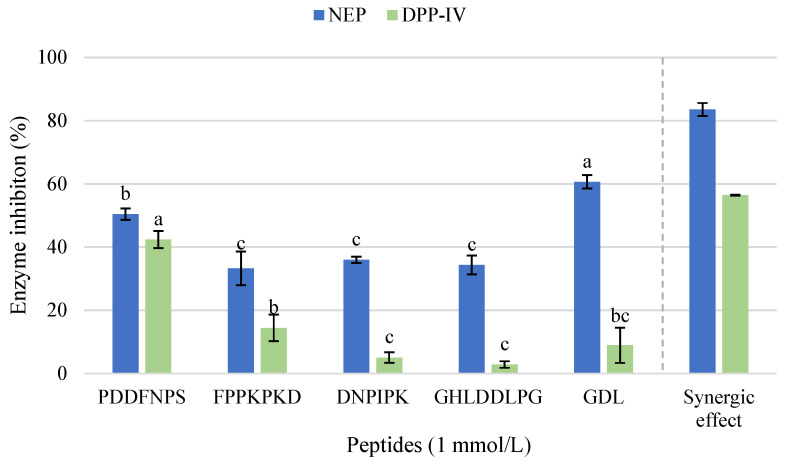
NEP and DPP-IV inhibitory activity of the five selected synthesized peptides PDDFNPS, FPPKPKD, DNPWIK, GHLDDLPG, and GDL (1 mmol/L) previously identified in fraction 1 (F1) by LC-MS/MS. Error bars mean standard deviation from triplicate replicates. Different letters between columns with the same color represent significant differences (*p* < 0.05).

Those peptides identified in F1 were subjected to in silico analysis using bioinformatics tools freely available online. Table 5 shows the most relevant sequences of peptides according to the results obtained using the Peptide Ranker tool (PPR) (ratio value > 0.5). Among these, the peptides PDDFNPS, FPPKPKD, DNPIPK, and GHLDDLPG and the tripeptide GDL were selected for synthesis and to confirm the previously detected bioactivity. The synthesized peptides showed no antioxidant activity or inhibitory capacity against TACE or MGL at 1 mmol/L. However, at this concentration, hypoglycemic activity mediated by inhibition of NEP or DPP-IV was observed (Figure 4). 

In terms of NEP inhibitory activity, all the peptides exhibited values above 30% at a concentration of 1 mmol/L. The tripeptide GDL stood out with a significantly higher inhibition value (60.65%), followed by the peptide PDDFNPS, which reached 50.42% of inhibition. The peptides FPPKPKD, DNPIPK, and GHLDDLPG showed NEP inhibition values between 33.28% and 35.99%, with non-significant differences between them. On the other hand, only the peptide PDDFNPS showed significant inhibition of DPP-IV, with a value of 42.38%. The obtained results suggested that PDDFNPS could be acting as a multifunctional inhibitor for both NEP and DPP-IV enzymes. In addition, a synergistic effect was observed when all the synthesized peptides were combined at a final concentration of 1 mmol/L. This mixture produced a higher inhibition of both enzymes compared to the individual peptides, reaching inhibition values of 83.54% for NEP and 56.43% for DPP-IV.

## 3. Discussion

Enzymatic hydrolysis has proved to be an effective process to obtain bioactive peptides and to release large amounts of amino acids. The presence of free amino acids in animal feed, particularly in aquaculture, is essential for the formation of proteins and hormones, as well as the regulation of numerous metabolic pathways related to animal growth and reproduction. Additionally, some of them can help against oxidative stress and reinforce the immune system [26,27]. For this reason, it is important to ensure a correct nutritional intake of both essential and non-essential amino acids [28,29]. In this context, it is important to highlight the high availability of essential amino acids shown in the PBSH, and the elevated presence of arginine, highly appreciated in aquaculture feed for its essential role in reproduction and correct endocrine regulation [30]. Furthermore, alanine (10.25%) and aspartic acid (4.6%) amino acids are also of great interest for aquaculture feed as they are the major glucogenic precursors in fish, as well as important energy substrates [28]. The presence of glutamine is also noteworthy as it is a key amino acid for cell growth, mainly in intestinal and immune cells, and, in addition, the presence of glutamic acid could have beneficial effects from the point of view of the immune system as it has been shown that this amino acid provides strong immunomodulatory effects [31]. Finally, leucine supplementation in the diet of rats with type II diabetes has been described to produce a significant reduction in blood glucose levels by exerting hypoglycemic activity [32].

Enzymatic hydrolysis to obtain antioxidant peptides has been extensively studied. In fact, the combination of the enzymes Alcalase and Protana Prime has been proved to be the most effective hydrolysis for the generation of antioxidant peptides in chicken feet hydrolysates in comparison with the combination of Alcalase and Flavourzyme [33]. Other porcine by-products have been subjected to several types of hydrolysis. In a screening of pork liver hydrolysates, López-Pedrouso et al. [34] reported the highest antioxidant activity values for ABTS (655.63 mg AA/100 g) and ORAC (22.64 mg Trolox/g) when Alcalase was used; however, the highest values in FRAP and DPPH were obtained in bromelain hydrolysates. These results were attributed to the effect of the abundant presence of some amino acids such as histidine, threonine, asparagine, serine, or taurine. In fact, some antioxidant peptides such as LEPVIGT and WGPGWE, obtained from porcine plasma after the hydrolysis with an alkaline protease and subsequent in vitro digestion, have been recently identified as showing protective effects against oxidative stress in HepG2 cells [35,36].

In relation to the stability of antioxidant peptides, despite the elimination of water during the freeze-drying process preventing bacterial growth, there may be some chemical degradation of the protein structures themselves over time. In fact, peptides can be degraded due to the action of different chemical mechanisms such as hydrolysis or oxidation, although peptide bonds scissions may have been favored by keeping the hydrolysate at room temperatures (around 20 °C), which favors the degradation of the peptide into smaller fragments, explaining the observed increase in antioxidant activity after six months of storage [37]. Regarding this, several authors have studied the effect of temperature on peptides. Liu et al. [38] reported an increase in antioxidant activity in insect hydrolysates (male silkmoth) in the ORAC assay when the temperature was raised from 25 °C to 60 °C and later decreased.

The presence of antioxidant peptides in PBSH is interesting as these peptides could also be used to delay or prevent oxidation of feed, as well as to prevent the appearance of pathological disorders associated with oxidative stress once the feed is ingested [39,40]. In fact, supplementation of feed with antioxidant compounds such as vitamin E has been described to improve metabolism in pigs and exert antioxidant and antimicrobial effect on meat by prolonging its shelf life after slaughtering, probably due to the protective effect of these compounds against oxidation [41]. Similarly, vitamins, minerals, and antioxidants are also commonly used in fish feed formulation, achieving excellent results in terms of performance, resistance to stress and disease, reduction of environmental impact, and maintenance of product quality [42,43].

The enzymes dipeptidyl peptidase 4 (DPP-IV) and neprilysin (NEP) are two key enzymes in glucose metabolism and the control of blood glucose levels. Both enzymes are capable of degrading glucagon-like peptide-1 (GLP-1), an incretin hormone responsible for stimulating insulin secretion. Recently, it has been shown that joint inhibition of both enzymes is more effective at keeping GLP-1 active for a longer period of time and improving insulin secretion [44]. PBSH hydrolysate has shown strong inhibitory power on DPP-IV and NEP enzymes. The neprilysin inhibitory activity has been poorly studied to date in peptide hydrolysates. Regarding this, pepsin and Flavourzyme hydrolysates of beef liver achieved inhibition rates close to 90% for NEP and between 55 and 60% for DPP-IV at 1 mg/mL and 10 mg/mL, respectively [45]. Similarly, ham bone hydrolysates prepared with different enzymes (Alcalase, Protamex, Protana Prime, and Flavourzyme) have also shown capacity to inhibit both enzymes, with the hydrolysate produced with Protamex being the most effective, reaching an inhibitory activity of 71% for NEP at a concentration of 9 mg/mL and 31.59% for DPP-IV at 8 mg/mL [46]. Although the inhibition of NEP by peptides from food by-product hydrolysates has not been extensively studied, over the last decade, the inhibition of the DPP-IV enzyme has been extensively studied, with numerous studies demonstrating that proteins derived from the milk of different species, such as casein or whey, can be effectively hydrolyzed to obtain DPP-IV-inhibiting peptides [47,48,49,50,51,52]. Indeed, recent studies have focused on marine by-products, including skin, heads, tails, bones, and viscera, as cost-effective sources of protein hydrolysates with similar inhibitory properties [53,54,55].

On the other hand, PBSH also showed a strong ability to inhibit TACE. The enzyme TACE (or ADAM17) is a membrane metalloprotease involved in numerous pathological processes in the body. Overexpression of this enzyme has been linked to various inflammatory diseases such as rheumatoid arthritis but also to cardiovascular diseases such as heart failure and even cancer [56]. Therefore, the presence of inhibitory peptides of this enzyme in the hydrolysate and, consequently, in feed could help to prevent the development of inflammatory processes in animals associated with biological or environmental factors or due to the management of the breeding facilities themselves. Although the effects of these TACE inhibitory peptides need to be studied in animals, recent studies have shown that the incorporation of different animal protein hydrolysates to feed improves immunity and survival in shellfish and aquaculture fish [57]. The fact that the peptides present in this hydrolysate also inhibit the enzyme monoacylglycerol lipase is of particular relevance. The MGL enzyme is a serine hydrolase that hydrolyses monoglycerides (MAGs), one of its most important substrates being 2-arachinononyl glycerol or (2-AG) due to its pharmacological importance, as well as its participation in the regulation of food intake. Thus, the action of MGL on 2-AG produces arachidonic acid, an important precursor of pro-inflammatory prostaglandins associated with metabolic, inflammatory, and neurodegenerative disorders and even related to the development of various types of cancer [58]. MGL inhibition has also been studied for its immunomodulatory effects as it is also involved in regulating the function of immune cells such as macrophages or neutrophils [59]. Indeed, a recent transcriptomic study in mouse cells has shown that MGL inhibition induces the expression of immune-related genes that trigger anti-inflammatory and neuroprotective effects [60]. In relation to this aspect, in the case of aquaculture, it is known that feed composition has a direct impact on numerous physiological processes within the organism, including the microbiota of the gastrointestinal tract of fish, which has also been proved to have a strong interconnection role with numerous other systemic pathways [61,62]. For this reason, the presence of functional ingredients in animal feed, which can provide bioactive anti-inflammatory and immunomodulatory peptides, is crucial for the correct development of the microbiota and so to enhancing animal health and welfare [63,64].

The identification and subsequent synthesis of several peptides led to the finding of five sequences with hypoglycemic potential through the inhibition of NEP and DPP-IV. The mechanisms involved in the inhibition of neprilysin are still not well-known; Heres et al. [65] reported values close to 20% of NEP inhibition for DG and EE dipeptides identified in dry-cured ham extract at a concentration of 1 mmol/L. However, a recent study with di- and tripeptides synthesized from chicken by-products has shown that the amino acid position within the peptide sequence is crucial for exhibiting higher NEP inhibitory activity. In fact, sequences rich in Val, Arg, Glu, Pro, or Ala showed a greater inhibitory effect, as well as those with an Arg or Glu residue at the C-terminal end [66]. In view of this, the presence of Pro in the synthesized peptides could be influencing their NEP inhibitory activity.

The DPP-IV inhibitory peptides’ characteristics have been extensively studied in recent years. Numerous peptides obtained from different protein sources such as milk (bovine, caprine, camel, or mare), marine sources (fish or seafood), meat sources and eggs, and vegetable or cereal sources have been reported as DPP-IV inhibitors [67]. The DPP-IV enzyme has a strict specificity, and its inhibition is strongly influenced by the type of amino acid and its position within the peptide chain. Thus, the presence of hydrophobic peptides can lead to an increase in DPP-IV inhibition [68]. In fact, NP, FN, and PS sequences within the PDDFNPS peptide have been reported to inhibit this enzyme according to the BIOPEP-UWM database.

This trend is also observed in other studies. The peptides GPF, IGL, and GGGW obtained by enzymatic hydrolysis of chicken blood with Alcalase and Protana Prime achieved inhibition values of the DPP-IV enzyme between 40.74 and 65.19% at a concentration of 2 mmol/L [69]. Other studies have used molecular docking tools to predict the molecular inhibitory interaction between the enzyme and the peptides. According to He et al. [70], potential peptides showing DPP-IV inhibitory activity were between four and seven amino acids in length after double hydrolysis of collagen (with papain and compound protease), with the GPVGPPG peptide having the highest molecular docking score and the highest content of conventional hydrogen bonds and therefore the highest inhibitory potential. Thus, the length of the PDDFNPS peptide as well as the presence of Pro as second residue of the C-terminal end (as in the GPVGPPG peptide) might have played a crucial role in exerting its inhibitory activity. Similarly, Wang et al. [71] reported the peptides GPAGPIGPV and GPAGPOGFPG obtained from the hydrolysis of sheepskin collagen and having Pro in the same position as potential DPP-IV inhibitors using molecular docking tools. In this sense, it was previously confirmed that a proline residue at the penultimate C-terminal position enhances DPP-IV inhibition by positioning the side chain in the P1 pocket of the enzyme [72]. The cyclic structure of proline fits tightly within the hydrophobic S1 site and restricts the peptide backbone to the favorable trans conformation, strengthening hydrophobic interactions and stabilizing the enzyme–peptide complex [71]. This structural feature accounts for the greater inhibitory potential of peptides having proline in this position.

## 4. Materials and Methods

### 4.1. Chemicals and Reagents

Alcalase 4.0 L and Protana™ Prime were purchased from Novozymes A/S (Novozymes A/S, Bagsvaerd, Denmark). Human Neprilysin Protein Tag free was obtained from Acro BioSystems (Acro BioSystems, Basel, Switzerland), and Aminopeptidase M from Merck (Merck, Darmstadt, Germany). Thiorphan, N-Succinyl-Ala-Ala-Phe-7-amido-4-methylcoumarin, HEPES, o-phthaldialdehyde (OPA), 2,2-diphenyl-1-picrylhydrazyl (DPPH), potassium ferricyanide, ferric chloride, (±)-6-hydroxy-2,5,7,8-tetramethylchromane-2 carboxylic acid (trolox), 2,2-azino-bis(3-ethylbenzothiazoline-6-sulfonic acid) diammonium salt (ABTS), and ascorbic acid were purchased from Sigma-Aldrich, Co. (St. Louis, MO, USA). Potassium persulfate and butylated hydroxytoluene (BHT) were purchased from Panreac Química S.A.U. (Panreac Química S.A.U., Barcelona, Spain). Peptides PDDFNPS, FPPKPKD, DNPIPK, GHLDDLPG, and GDL were synthesized at Universitat Pompeu Fabra (Universitat Pompeu Fabra, Barcelona, Spain). All other chemicals and reagents were of analytical grade.

### 4.2. Preparation of Porcine Blood Spray-Dried Hydrolysate (PBSH)

Fresh liquid porcine blood was collected under hygienic conditions during the slaughter process and stored at refrigerated temperature. Then, porcine blood was diluted 1:1 with bidistilled water and subjected to ultrasound pretreatment (1 h, 35 kHz) (Transsonic TI-H, VWR, Elgin, IL, USA). The protein content of the blood was determined by the Dumas method [73], resulting in a total amount of 21%. Then, enzymatic hydrolysis was carried out in a 1 L Premium Minireactor (Scharlau, Sentmenat, Barcelona, Spain) with 2% Alcalase 4.0 L for 2 h at 65 °C, followed by 5% Protana™ Prime for 16 h at 55 °C. Later, enzymes were inactivated by heating at 85 °C for 15 min. After hydrolysis treatment, the hydrolysate was subjected to membrane ultrafiltration through 10 kDa filters (Ultracel^®^ 10 kDa Ultrafiltration Discs, EMD Millipore Corporation, Billerica, MA, USA) under pressure conditions with N_2_ (Air Liquide, Madrid, Spain) using 200 mL Amicon^®^ Stirred Cell containers (EMD Millipore Corporation, Billerica, MA, USA). Finally, the hydrolysate was spray-dried using a Mini Spray Dryer S-300 (BUCHI Iberica S.L.U, Sant Just Desvern, Barcelona, Spain).

### 4.3. Determination of Free Amino Acids Content (FAAs)

The determination of FAAs was performed following the method described by Aristoy & Toldrá [74]. First, the sample (in triplicate) was prepared by diluting the spray-dried hydrolysate with bidistilled water at the adequate concentration for using 5 mmol/L of norleucine as an internal standard. After amino acid derivatization, the chromatographic separation was carried out at 52 °C and a flow of 1 mL/min using a reverse-phase HPLC chromatograph with a Waters Pico Tag^®^ C18 column (3.9 × 300 mm; Waters Corp., Milford, MA, USA). The elution was measured at 254 nm. Phase A was composed of 70 mmol/L sodium acetate with 2.5% acetonitrile, and phase B was prepared by the mixture of acetonitrile (45%), bidistilled water (40%), and methanol (15%). Finally, the results were as mg of FAAs/g of PBSH.

### 4.4. ABTS Radical-Scavenging Activity

The ABTS assay was performed in triplicate following the protocol outlined by Gallego et al. [75]. ABTS+ solution was diluted with 50 mmol/L phosphate-buffered saline (PBS) at pH 7.4 until the absorbance reached 0.70 at 734 nm, measured with a UV-Visible spectrophotometer (Cary 60 UV-visible spectrophotometer, Agilent Technologies, Santa Clara, CA, USA). Following this, 10 μL of sample (10 mg PBSH/mL water) was added to 990 μL of the ABTS+ solution, and the absorbance was measured at 734 nm after a 6 min incubation in darkness. Ascorbic acid was used as a positive control and PBS as a negative control. The antioxidant capacity of the hydrolysate was reported as μmol TEAC (Trolox equivalent antioxidant capacity/mL), calculated from a standard curve of Trolox.

### 4.5. Ferric Reducing Antioxidant Power (FRAP)

The FRAP assay was carried out in triplicate by mixing 70 μL of sample (20 mg PBSH/mL water) with an equal volume of 200 mmol/L phosphate buffer (pH 6.6) and potassium ferricyanide (10 mg/mL), incubated at 50 °C for 20 min. After adding 70 μL of trichloroacetic acid (100 mg/mL), the mixture was centrifuged at 200× *g* and 25 °C for 10 min. Then, 140 μL of the supernatant was combined with 140 μL of distilled water and 28 μL of ferric chloride (1 mg/mL) in a 96-well transparent microplate, followed by 10 min of incubation at room temperature in darkness. Absorbance was measured at 700 nm using a CLARIOstar microplate reader (BMG Labtech, Ortenberg, Germany). For the controls, PBS was the negative and BHT (2 mg/mL in ethanol) the positive. Results were expressed as TE (Trolox equivalents) per mL, calculated from a Trolox standard curve.

### 4.6. DPPH Free Radical-Scavenging Activity

DPPH activity was determined by adding 500 μL of ethanol and 125 μL of DPPH solution (0.02% DPPH in ethanol) to 100 μL of sample in a concentration of 20 mg PBSH/mL water in triplicate. After 1 h of incubation avoiding light exposure, absorbance was measured at 517 nm. BHT (20 mg/mL) was used as a positive control.

### 4.7. Study of the Antioxidant Activity Stability

The spray-dried porcine blood hydrolysate (PBSH) was kept for 6 months under refrigerated conditions (4 °C) and at room temperature (21 °C) to study the stability of the antioxidant activity at two different storage conditions. The antioxidant activity assays were performed once a month for six months.

### 4.8. Determination of Dipeptidyl Peptidase IV (DPP-IV) Enzyme Inhibition

DPP-IV inhibitory activity was determined using “DPP-IV inhibitor screening kit” (MAK203) (Sigma-Aldrich, St. Louis, MO, USA) according to the manufacturer instructions. PBSH samples were assayed at 20 mg/mL. The fluorescence (FLU, λex = 360/λem = 460) was measured in a CLARIOstar microplate reader (CLARIOstar, BMG Labtech, Germany) in kinetic mode during 30 min at 37 °C, and readings were taken every minute. Sitagliptin was used as positive control, and bidistilled water as a negative one.

### 4.9. Determination of Neprilysin (NEP) Enzyme Inhibition

The NEP inhibitory activity was performed as described in [66]. Briefly, 25 μL of sample (20 mg PBSH/mL water) in triplicate was mixed with 25 μL of enzyme solution prepared by diluting NEP stock (400 µg/mL) 1:1000 with bidistilled water. After an incubation of 10 min at 37 °C, 50 μL of substrate solution was added in each well. The substrate solution consisted of 0.2 mmol/L N-Succinyl-Ala-Ala-Phe-7-amido-4-methylcoumarin (AMC) in 50 mmol/L HEPES/NaOH buffer (pH 7.4) with the presence of 0.242 µL/well (0.75 µg/well) of aminopeptidase M. Thiorphan (1 mmol/L) was used as a positive control, and bidistilled water as a negative. Fluorescence was monitored at Ex/Em = 320/420 nm during 60 min at 37 °C in a CLARIOstar microplate reader (CLARIOstar, BMG Labtech, Germany). For this assay, HPLC fractions were diluted 1:10 from the resuspended samples.

The inhibition percentage was calculated with the following equation, choosing two times in a linear range (ΔF = T2 – T1):

(1) $$\;\%Inhibition=\;\frac{∆F\;of\;enzyme\;control-\;∆F\;of\;sample\;}{∆F\;of\;enzyme\;control}\;\times \;100$$

where F means Fluorescence, T1 is the initial time, and T2 is the final time of the linear range.

### 4.10. Determination of Tumor Necrosis α-Converting Enzyme (TACE) Inhibition

The inhibition of TACE enzyme was determined using the kit “TACE Inhibitor Screening Assay Kit” (MAK218) (Sigma-Aldrich, St. Louis, MO, USA). PBSH was assayed in triplicate at 20 mg/mL; GM6001 was used as a positive control, and bidistilled water as a negative. Fluorescence (Ex/Em = 318/449 nm) was measured during 30 min at 37 °C in a CLARIOstar microplate reader (BMG Labtech, Germany). All reagents and calculations were carried out according to the protocol of the kit.

### 4.11. Determination of Monoacylglycerol Lipase (MGL) Inhibition

The fluorometric “Monoacylglycerol Lipase/MGL Inhibitor Screening kit” (ab283388) (Abcam, Cambridge, UK) was used to determine MGL inhibition. All the reagents were prepared according to the manufacturer instructions. PBSH was assayed in triplicate at 20 mg/mL; JJKK-048 was used as a positive control, and bidistilled water as a negative. The fluorescence (Ex/Em = 360/460 nm) was measured for 60 min at 37 °C in a CLARIOstar microplate reader (CLARIOstar, BMG Labtech, Germany).

### 4.12. Study of Peptide Profile by RP-HPLC Separation

The spray-dried hydrolysate was fractionated according to hydrophobicity using reverse-phase liquid chromatography (RP-HPLC). The separation was performed on an Agilent 1100 Series chromatograph with a C18 column (4.6 × 250 mm, 5 µm, Waters Co., Milford, MA, USA) in gradient mode. Two phases were prepared: phase A consisted of distilled water with 0.1% TFA, and phase B contained 60% acetonitrile and 40% distilled water, with 0.085% TFA. A 100 µL sample was injected at a concentration of 40 mg/mL. During the first two minutes, 100% of phase B was used, followed by a gradient up to 50% to complete the chromatogram after 50 min. Fractions were collected every 10 min and lyophilized. The samples were resuspended in 500 µL of bidistilled water for subsequent assays.

### 4.13. Peptide Identification by Mass Spectrometry in Tandem

Peptide sequence identification was performed on a Tims TOF fleX tandem mass spectrometer (Bruker, Bremen, Germany). The sample was loaded onto an Evotip and then eluted on an analytical column (PepSep 8 cm × 100 µm, 3 µm; Evosep, Odense, Denmark) in an Evosep One system, using the 30 SPD chromatographic method defined by the manufacturer. The eluted peptides were ionized by electrospray at 1700 V and 200 °C and analyzed in data-dependent acquisition mode (DDA PASEF) under the following conditions: TIMS conditions were in random mode, 1/K0: 0.7–1.76 V·s/cm^2^; ramp time: 100 ms; Duty Cycle: 100%; Ramp Rate: 9.42 Hz; MS Averaging: 1; Auto Calibration: off. MS1 scans were obtained from 100 to 1700 *m*/*z* in positive ionization mode and PASEF Scan Mode. For MS2 experiments, up to 10 PASEF ramps were acquired over 1.7 s in high-sensitivity mode, with no minimum charge and a maximum charge of 3. System sensitivity was controlled using 50 ng of a HELA protein hydrolysate. For peptide identification, the acquisition of data and generation of peak lists were carried out using Mascot Distiller software (Matrix v2.8, Science, Inc., Boston, MA, USA) including the following conditions: taxonomy Chordata, non-enzyme specified, peptide mass tolerance of 100 ppm, and fragment mass tolerance of 0.3 Da.

### 4.14. In Silico Analysis of the Identified Peptides

The Peptide Ranker tool (http://distilldeep.ucd.ie/PeptideRanker/, accessed on 21 November 2024) was used to predict the potential bioactivity of the identified peptides of each fraction. Peptides with values close to 1 have a higher probability of being bioactive.

### 4.15. Statistical Analysis

Rstudio v.4.3.3 software (R Foundation for Statistical Computing, Vienna, Austria) was used to perform all the statistical analyses. The data were analyzed using a one-way or two-way ANOVA, followed by a Tukey’s Honest Significant Difference (HSD) test (*p*-value ≤ 0.05).

## 5. Conclusions

PBSH hydrolysate enabled the sustainable valorization of porcine blood through sequential enzymatic hydrolysis. The in vitro study of the bioactivity revealed antioxidant, hypoglycemic, anti-inflammatory, and immunomodulatory effects attributed to the peptides present in PBSH. After the fractionation of the extract, the most polar fraction exhibited the highest bioactivity, showing the greatest inhibition of NEP, DPP-IV, TACE, and MGL enzymes. Subsequently, five peptide sequences associated with hypoglycemic activity were identified, showing synergistic effects among them at a concentration of 1 mmol/L. The peptide PDDFNPS stood out for its multifunctional nature, exerting dual inhibition of DPP-IV and NEP with percentages of inhibition of 56.43 and 83.54%, respectively. These findings position PBSH hydrolysate as a promising alternative for its inclusion in aquaculture feed, being able to contribute not only to the sustainability of food and aquaculture sectors but also to animal welfare. However, further studies are necessary to confirm the in vivo effect, large-scale performance, and the stability when it is incorporated in animal feed.

## Figures and Tables

**Table 1:** Biological activities of the spray-dried porcine blood hydrolysate (PBSH). Values shown are means ± standard deviation of three replicates.

Biological Activity	Assay	Concentration (mg/mL)	Results	IC_50_ (mg/mL)
Antioxidant	ABTS (µmol TEAC/g)	10	2048.48 ± 20.01	2.09
	FRAP (µmol TE/g)	20	589.99 ± 59.64	135.05
	DPPH (%)	20	35.94 ± 4.86	26.73
Hypoglycemic	Dipeptidylpeptidase-IV (DPP-IV) inhibition (%)	20	82.78 ± 1.55	-
	Neprilysin inhibition (%)	20	84.72 ± 1.14	-
Anti-inflammatory	TACE inhibition (%)	100	50.79 ± 2.24	-
Immunomodulatory	Monoacylglycerol lipase (MGL) inhibition (%)	20	69.08 ± 6.93	-

**Table 2:** Stability of the antioxidant activity of the porcine blood spray-dried hydrolysate (PBSH) stored under refrigeration (4 °C) and at room temperature (20 °C). Values shown are means ± standard deviation of three replicates. Means in the same row without a common letter in superscript are significantly different (*p* < 0.05).

Antioxidant Activity	T (°C)	Storage Time (Months)					
		1	2	3	4	5	6
ABTS (µmol TEAC/g)	4 °C	2052.0 ± 15.3 ^a^	2123.5 ± 12.9 ^b^	2014.7 ± 4.2 ^b^	1917.3 ± 14.5 ^c^	1946.0 ± 13.8 ^c^	2042.3 ± 16.5 ^b^
	20 °C	2116.2 ± 16.9 ^a^	2057.6 ± 30.1 ^b^	2030.5 ± 17.8 ^b^	1926.0 ± 12.5 ^c^	2017.8 ± 8.4 ^b^	2056.1 ± 6.6 ^b^
FRAP (µmol TE/g)	4 °C	414.6 ± 16.5 ^c^	306.3 ± 7.2 ^d^	333.3 ± 20.6 ^d^	345.9 ± 7.2 ^d^	329.9 ± 20.8 ^d^	318.6 ± 4.6 ^d^
	20 °C	307.3 ± 32.3 ^d^	515.6 ± 26.0 ^b^	478.3 ± 34.2 ^b^	643.3 ± 9.0 ^a^	635.3 ± 28.4 ^a^	535.3 ± 28.4 ^b^
DPPH (% inhib)	4 °C	30.5 ± 1.4 ^g^	30.7 ± 0.7 ^g^	30.1 ± 2.3 ^g^	32.0 ± 3.9 ^fg^	32.0 ± 2.1 ^fg^	37.1 ± 2.1 ^ef^
	20 °C	40.3 ± 1.5 ^e^	40.6 ± 2.4 ^e^	46.3 ± 0.6 ^d^	51.9 ± 0.9 ^c^	59.2 ± 0.5 ^b^	72.3 ± 0.9 ^a^

**Table 3:** Free amino acid composition (mg/g) of the spray-dried porcine blood hydrolysate (PBSH). Values shown are means ± standard deviation of three replicates.

Free Amino Acids	Concentration
	(mg/g)
Aspartic Acid (Asp)	21.33 ± 0.88
Glutamic Acid (Glu)	19.88 ± 0.75
Hydroxyproline (Hyp)	0.08 ± 0.01
Serine (Ser)	12.44 ± 0.28
Asparagine (Asn)	23.40 ± 0.62
Glycine (Gly)	16.24 ± 0.57
Glutamine (Gln)	5.39 ± 0.20
β-Alanine (βAla)	0.33 ± 0.01
Taurine (Tau)	0.33 ± 0.01
Histidine (His)	40.11 ± 1.58
γ-Aminobutyric acid (γAba)	1.12 ± 0.09
Threonine (Thr)	17.29 ± 0.56
Alanine (Ala)	47.35 ± 1.43
Arginine (Arg)	6.86 ± 0.18
Proline (Pro)	4.43 ± 0.12
Tyrosine (Tyr)	5.07 ± 0.11
Valine (Val)	49.17 ± 1.27
Methionine (Met)	5.92 ± 0.16
Cysteine (Cys)	1.28 ± 0.05
Isoleucine (Ile)	3.65 ± 0.10
Leucine (Leu)	82.04 ± 2.08
Phenylalanine (Phe)	33.70 ± 0.82
Tryptophan (Trp)	12.28 ± 0.34
Ornithine (Orn)	4.84 ± 0.33
Lysine (Lys)	47.28 ± 2.05
Total	461.82 ± 14.13

**Table 4:** Biological activities of the peptide fractions obtained by RP-HPLC. Values shown are means ± standard deviation of three replicates. Means in the same row without a common letter in superscript are significantly different (*p* < 0.05).

Biological Activity	Assay	Fraction				
		F1	F2	F3	F4	F5
Antioxidant	ABTS (µmol TEAC/g)	224.28 ± 2.13 ^d^	1441.31 ± 15.14 ^a^	1397.22 ± 12.06 ^b^	494.39 ± 6.92 ^c^	336.87 ± 22.63 ^d^
	FRAP (µmol TE/g)	89.94 ± 1.90 ^a^	11.18 ± 4.97 ^b^	n.d.	n.d.	11.59 ± 1.43 ^b^
	DPPH (% inhibition)	17.01 ± 2.81 ^a^	7.16 ± 1.25 ^bc^	8.50 ± 2.12 ^b^	6.59 ± 0.52 ^bc^	3.50 ± 0.94 ^c^
Hypoglycemic	DPP IV inhibition (%)	90.45 ± 1.16 ^a^	n.d.	n.d.	40.29 ± 3.99 ^b^	n.d.
	NEP inhibition(%)	84.69 ± 1.68 ^a^	49.67 ± 0.53 ^b^	55.09 ± 3.78 ^b^	30.43 ± 2.83 ^c^	36.54 ± 3.92 ^c^
Anti-inflammatory	TACE inhibition (%)	113.23 ± 5.10 ^a^	37.04 ± 2.42 ^b^	23.81 ± 4.49 ^c^	8.73 ± 1.12 ^d^	14.29 ± 2.24 ^cd^
Immunomodulatory	MGL inhibition (%)	n.d.	n.d.	n.d.	85.30 ± 3.61	n.d.

n.d.: non detected activity.

**Table 5:** Sequences of the most relevant peptides identified in the first fraction (F1) of PBSH hydrolysate collected from RP-HPLC.

Source Protein	Protein ID	Peptide Sequence	Peptide Ranker Ratio ^1^
Albumin	ALBU_PIG	DNPDIPK	0.552992
		DNPDIPKLKPDP	0.501574
		EDEQKFWGK	0.570703
		DNPDIPKLKPDPV	0.523743
Hemoglobin A	HBA_PIG	GHLDDLPGA	0.535194
		HPDDFNPS	0.575335
		GHLDDLPG	0.588214
		PDDFNPS	0.588007
		HHPDDFNPS	0.529012
		AHHPDDFNPS	0.517146
		GHLDDLPGAL	0.698265
Hemoglobin B	HBB_PIG	DPENFRL	0.774659
Immunoglobulin	IGHG1_HUMAN	FPPKPKD	0.625664
		GDL	0.546664

^1^ The Peptide Ranker tool was used to predict the potential bioactive peptides. Scores closer to 1 indicate higher probability that the peptide will be bioactive.

## Data Availability

The original contributions presented in this study are included in the article. Further inquiries can be directed to the corresponding authors.
